# Effect of a Selective Progesterone Receptor Modulator on Induction of Apoptosis in Uterine Fibroids In Vivo

**DOI:** 10.1155/2012/436174

**Published:** 2012-07-16

**Authors:** Petr Horak, Michal Mara, Pavel Dundr, Kristyna Kubinova, David Kuzel, Robert Hudecek, Roman Chmel

**Affiliations:** ^1^Department of Obstetrics and Gynaecology, First Faculty of Medicine, Charles University in Prague, Apolinarska 18, 128 00 Prague, Czech Republic; ^2^Institute of Pathology, First Faculty of Medicine, Charles University in Prague, 128 00 Prague, Czech Republic; ^3^Department of Obstetrics and Gynaecology, University Hospital Brno, 625 00 Brno, Czech Republic; ^4^Department of Obstetrics and Gynaecology, Second Faculty of Medicine, Charles University in Prague, 150 06 Prague, Czech Republic

## Abstract

*Aim*. To determine if hormonal treatment induces apoptosis in uterine fibroids. *Methods*. Immunohistochemical examination of fibroid tissue, using avidin-biotin complex and cleaved caspase-3 antibody for detecting apoptosis, was performed in premenopausal women who underwent 12-week treatment with oral SPRM (6 patients with 5 mg and 5 patients with 10 mg of ulipristal acetate per day) or gonadoliberin agonist (GnRHa, 17 patients) and subsequent myomectomy or hysterectomy for symptomatic uterine fibroids. Ten patients with no presurgical hormonal treatment were used as controls. *Results*. Apoptosis was present in a significantly higher proportion of patients treated with ulipristal acetate compared to GnRHa (*P* = 0.01) and to patients with no hormonal treatment (*P* = 0.01). In contrast to an AI of 158.9 in SPRM patients, the mean AI was 27.5 and 2.0 in GnRHa and control groups, respectively. No statistical difference in the AI was observed between the two groups of patients treated with ulipristal acetate (5 mg or 10 mg). *Conclusion*. Treatment with ulipristal acetate induces apoptosis in uterine fibroid cells. This effect of SPRM may contribute to their positive clinical effect on uterine fibroids.

## 1. Introduction

Uterine fibroids are the most common benign gynecological tumors. Their prevalence in premenopausal patients is 30–40%, making them one of the most common reasons for gynecological surgery [1].

The etiology and pathophysiology are still unknown. It is considered that various genetic, anthropometric, racial, reproductive, and vascular factors, as well as the role of growth factors or some hormones, particularly ovarian steroids, could play a role [2–7]. 

Surgery still dominates fibroid treatment; the most common is myomectomy or hysterectomy depending on the age and reproductive status of the patient. Nowadays, pharmacological intervention is used as a symptomatic therapy in smaller fibroids. There are few drugs that have the potential to have a direct effect on fibroid growth. The most promising in this category are the selective progesterone receptor modulators (SPRMs) that have become the subject of intense investigation in recent years but have not yet been introduced into standard clinical practice. The mechanism of action of these drugs is still unknown but the effect on myoma-related symptoms and fibroid shrinkage was proven in early clinical studies [8, 9]. Their selective antiproliferative, proapoptotic, and antifibrinolytic effect on fibroids has been demonstrated in human tissue cultures *in vitro *[10]. The aim of our study was to determine the eventual higher apoptosis rate in fibroids extirpated from patients given SPRM pretreatment compared with controls. 

## 2. Materials and Methods 

### 2.1. Patient Recruitment

Patients with uterine fibroids were given ulipristal acetate (PGL4001) during 12 weeks prior to the planned surgery. This drug, provided by PregLem S.A., Switzerland, belongs to the SPRM group. These patients had participated in a phase III clinical study with ulipristal acetate. This study, in which patients received daily dose of 5 or 10 mg of ulipristal acetate or placebo, evaluated the efficacy of ulipristal acetate on symptomatic uterine fibroids [11]. Patients that received active treatment and required surgery for their fibroids were included in the present study.

For a comparable group, we chose patients with symptomatic fibroids who were treated with gonadoliberin agonist (GnRHa) triptorelin (Ipsen Pharma Biotech, France) 12 weeks prior to planned surgery at a dose of 3 mg intramuscularly 3 times at 28-day intervals. For controls we enrolled patients with the same diagnosis who were receiving no hormonal pretreatment and were referred to have either a hysterectomy or myomectomy. The operation was always scheduled within 2 weeks from the last SPRM dose or 6 weeks within the last dose of GnRHa.

Our study did not have a randomized or double-blind design. Patient recruitment into each group was dependant on a patient informed choice. In order to reduce anemia and the risk of perioperative blood transfusion and if patients met the inclusion criteria (see below), they were given the option of 12 weeks of hormonal pretreatment with ulipristal acetate (as part of the multicenter placebo-controlled study with SPRM) or triptorelin. If the patients preferred early surgery with no hormonal treatment, they were included in the control group. Patients were fully informed of all known advantages, disadvantages, and differences between the two options of treatment including the fact that if patients choose the SPRM, they could be randomized into the placebo subgroup. 

All the patients in the study were administered oral iron supplements (Ferrous sulphate 80 mg once daily) starting either with the administration of hormonal treatment (both groups with hormonal pretreatment) or on the day of the enrolment in the study (control group).

### 2.2. Inclusion and Exclusion Criteria

Patients aged between 18 and 50 years of age with uterine fibroid/s sized ≥3 cm (the largest measurable diameter of myoma measured by vaginal ultrasonography prior to ulipristal or triptorelin administration or before surgery in the control group), typical myoma-related symptoms (menorrhagia with PBAC (Pictorial Bleeding Assessment Chart) score higher than 100 for the 1st–8th days of menstruation; eventually pressure pelvic symptoms) [12, 13] and significant anemia (hemoglobin ≤ 100 g/L) were included in the study.

Exclusion criteria were patients with an overall size of uterus exceeding 16th week of pregnancy, history of uterine surgery, hormonal supplement therapy and hormonal contraception administration or other hormonal treatment with estrogen or progesterone within the last month prior to the study, BMI ≤ 18 or ≥40, hemoglobinopathy, atypical hyperplasia or endometrial carcinoma, cervical cancer, ovarian or breast cancer, endometrial polyp larger than 2 cm, and ovarian cyst larger than 4 cm. Patients who refused to sign the informed consent and patients who, regardless of the reasons, wanted to terminate their participation in the study were also excluded. We also excluded women whose histological examination of the extirpated tumor of the uterus or the entire uterus brought a different result than vital (therefore evaluable) leiomyoma and patients (from the study of PregLem S.A.) that used placebo instead of ulipristal acetate.

The study protocol was approved by the Ethics Committee of the first Medical Faculty of Charles University in Prague. All patients enrolled signed an informed consent.

### 2.3. Laboratory Examination of Fibroids, and Statistical Analysis

All study women who underwent myomectomy (open or laparoscopic) or hysterectomy (laparoscopically assisted vaginal or open) were subject to standard histological examination of the removed myoma or the entire uterus and immunohistochemical tests to detect apoptosis. These examinations were performed and evaluated by the same pathologist who was not informed of whether the patient received any hormonal therapy prior to the surgery.

Immunohistochemistry was performed using the avidin-biotin complex method with antibody against cleaved caspase-3 (dilution 1 : 250, Cell Signaling Technology, Beverly, MA). Antigen retrieval was performed with a sodium citrate buffer (pH 6.0) in a water bath for 40 minutes. The apoptotic index (AI), the number of apoptotic cells of all cells counted, was determined manually using an ocular counting grid at three randomly chosen fields. One thousand cells for each sample were counted [14, 15].

We used a nonparametric Kruskal-Wallis test and Mann-Whitney test (with the Bonferroni adjustment of *P* values for multiple testing) for statistical comparison of the results of the AI between the subgroups (SPRM versus GnRHa, SPRM versus controls, GnRHa versus controls, and SPRM 10 mg versus SPRM 5 mg). For comparison between patients with an AI higher than 10, a chi-square test and Fisher test were used.

## 3. Results

A total of 41 symptomatic patients who met the inclusion criteria were recruited to our study between November 2008 and December 2009. Out of these 41 patients, 17 patients preferred GnRHa and 10 patients requested an early operation with no pretreatment. In the placebo-controlled study with ulipristal acetate 14 patients were included, of which 3 patients were subsequently excluded following the unblinding of data revealing placebo administration. Uterine fibroids from the remaining 38 patients were examined histologically after surgery and immunohistochemical tests were performed to detect apoptosis. The baseline characteristics of each group are summarized in Table 1. Patients in each group did not significantly differ in age, BMI, size of dominant fibroid or parity. All the histological findings revealed conventional leiomyomata.

**Figure 1:**
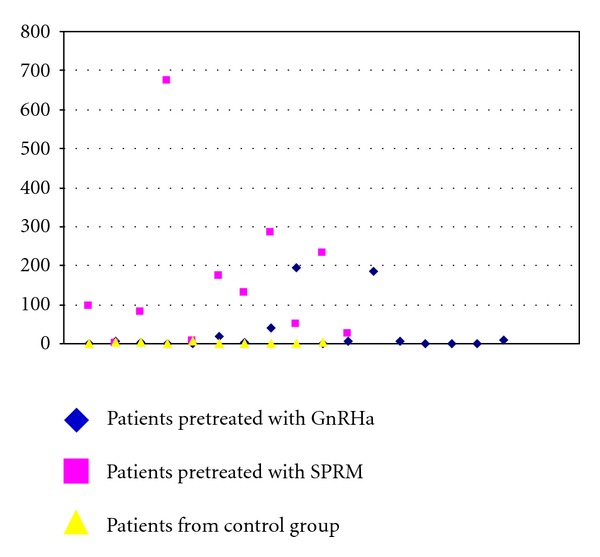
Apoptotic index in separate groups.

Based on the results of immunohistochemistry, an AI was calculated for each patient. Zero or minimal percentage (less than 1% of 1000 cells examined) of cells with apoptosis were detected in 18.2% of patients receiving preoperative SPRM compared to 76.5% of patients receiving GnRHa and 100% in the control group. The differences between SPRM versus GnRHa (*P* = 0.01, *χ* ^2^ test) and SPRM versus controls (*P* = 0.001, Fisher's test), respectively, were statistically significant (see Table 2). The highest average AI value was described in the SPRM group (157.9), which was significantly higher than that in GnRHa group (27.5; *P* = 0.01) and the control group (2.0; *P* = 0.01). The results of each single patient including detailed comparison of the subgroups are shown in Figure 1 and Table 2.

We also tried to determine if there was any difference in the incidence of apoptosis between patients with different doses of ulipristal as well as if a dose-dependent apoptosis rate in the SPRM group could be found (Table 3). In our limited group of 11 patients, we did not observe a higher proportion of cells with apoptosis in women receiving a higher dose of SPRM (*P* = 0.144, Mann-Whitney test).

## 4. Discussion

Uterus-sparing therapy remains an up-to-date topic even in cases of women no longer desiring pregnancy. Myomectomy remains the most frequently used surgical technique. There is a constant search for alternatives to myomectomy because this operation is both quite invasive for the patient and risky and devastating for the uterus before planned pregnancy. Apart from occlusive methods aimed at fibroid devascularization (uterine artery embolization (UAE), and laparoscopic uterine artery occlusion (LUAO)) new modalities such as thermoablation of fibroids by focused ultrasound or radiofrequency ablation are beginning to be used [16–19].

None of the above-stated methods, however, affect pathophysiology of fibroids or have systematic effects. Some hormonal drugs have the potential to treat the cause of the disease. Many drug groups, such as progesterone (including intrauterine application), Danazol, gonadoliberin agonists and antagonists, selective estrogen receptors modulators, aromatase inhibitors, or antiprogesterone, have been used in this indication [20–25]. None of the drugs have made a significant breakthrough in fibroid treatment.

GnRHa is the most used and studied to date. These drugs cause hypoestrinism, which, after several months of use, leads to a slight fibroid volume reduction [15, 26]. The use of GnRHa is unfortunately accompanied with a number of drawbacks, which is why their use in the treatment of fibroids has been limited to three-month pretreatment in selected patients before myomectomy or hysterectomy [27–29].

Fibroid cells demonstrate higher concentration of estrogen receptors compared to surrounding myometrium, equally higher expression of mRNA and of progesterone receptors (PRs) A and B. Increase of mitotic activity and fibroid size in the secretory phase of the cycle has also been described [30–32]. Therefore, the logical effort is to use drugs not only causing hypoestrinism but also affecting PR. In addition to antiprogesterone, Mifepristone, an SPRM, may become one of the drugs used in fibroid treatment [33, 34], due to their targeted mode of action. Unlike Mifepristone, which apart from reduction of menorrhagia and reduction of uterine and fibroid volume leads to hyperplasia of the endometrium, SPRM with its modified both agonistic and antagonistic PR effect does not have this undesirable effect on the endometrium [35, 36]. SPRMs act directly on the endometrium by maintaining its glandular and stromal proliferation at low levels and thus causing amenorrhea in most of the patients without causing hypoestrinism. Similar morphological changes, as well as a reduction in mitotic activity, were detected in cells of leiomyomas examined after hysterectomy of 33 patients receiving 12-weeks treatment with asoprisnil prior to the surgery [37].

In cell cultures SPRMs lead to reduction of cell viability, suppression of expression of growth factors and induced apoptosis through mitochondrial activation and tumor necrosis factor-related apoptosis inducing ligand (TRAIL) [38]. Bcl-2 is considered to be the key protein in the inhibition of apoptosis. It has been demonstrated that a Bcl-2 promoter interacts with PR by progesterone, suggesting in what other ways SPRMs may induce apoptosis [39]. But the proapoptotic effect of an SPRM has not yet been verified in vivo. Asoprisnil (J867) is a typical example of the SPRM, which selectively induces apoptosis in leiomyomas cells in tissue cultures without causing proliferation or apoptosis in normal cells of the myometrium [10, 40]. In our study, we used ulipristal acetate (PGL 4001 or CDB-2914, 17alpha-Acetoxy-11beta-(4-N, N-dimethylaminophenyl)-19-norpregna-4,9-diene-3,20-dione), a steroid substance reversibly blocking progesterone receptors. Despite the limited number of patients in the study we observed a significantly higher apoptosis rate in fibroid cells exposed to ulipristal acetate preoperatively compared to fibroids of patients treated with GnRHa as well as in fibroids with no preoperative hormonal treatment. Apoptosis may thus be an important, although apparently not the only, mechanism of an SPRM suppression effect on uterine fibroids. The other factor may be, for instance, uterine artery flow reduction [9]. However, our results should not be generalized to all preparations that modify PR because the group of SPRMs seems to be heterogeneous. The fact that the apoptosis rate was not significantly higher using twice the daily dose of ulipristal acetate (10 mg) suggests that a daily dose of 5 mg ulipristal acetate is sufficient for apoptosis induction and a higher dose apparently does not increase its proapoptotic effect. Unfortunately, at the moment we cannot say if longer use of ulipristal acetate like 6 or 12 months could lead to even larger proapoptotic effect in fibroids or not.

**Figure 2:**
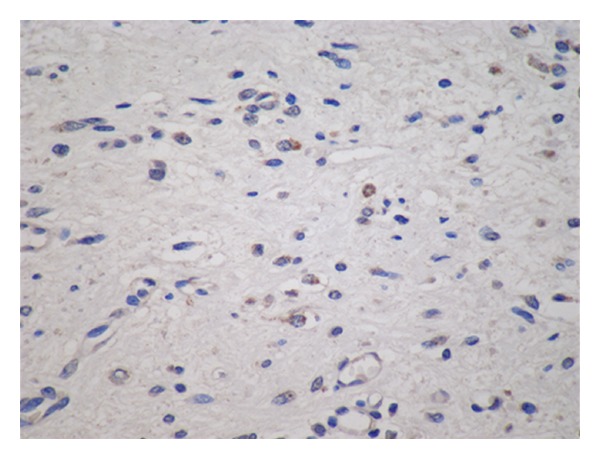
Immunohistochemical analysis using cleaved caspase-3 antibody. Note the granular cytoplasmic positivity in apoptotic cells.

Apoptosis is one of the main types of programmed cell death. It incorporates a series of biochemical processes leading to typical changes in cell appearance. This process is then followed by removal of the cells (without inflammation), making apoptosis in the foundations different from necrosis. The borderline between apoptosis and necrosis is however not clear and sometimes both processes combine making the designation of the cell death ambiguous [41]. The following laboratory methods are used in apoptosis detection: phosphatidylserine-annexin V, DNA fragmentation (ELISA), Laddering, TUNEL (terminal deoxynucleotidyl transferase dUTP nick end labeling), Fas, TNFR1, and P53 [42]. In our study, we used the method of indirect immunohistochemistry with cleaved caspase-3 antibody (Figure 2). We used an avidin-biotin complex (ABC method) technique with visualization using horseradish peroxidase and diaminobenzidine.

Selective proapoptotic and antiproliferative effects of SPRM preparations could be the ideal mechanism for suppression of uterine fibroids with a permanent or at least longer effect compared to GnRHa without adverse events of hypoestrinism, concurrently with much safer results than the necrosis caused by the UAE. Their big clinical potential (cessation of excessive uterine bleeding, correction of severe anemia, and volume reduction of the fibroids and the uterus) and safety in women with fibroids have been recently proved by two large randomized trials [11, 43]. On the other hand the necrosis after UAE, which can also occur inside the uterine cavity, can greatly reduce the chances for patients to have a successful pregnancy and that is why UAE is considered to be relatively contraindicated in patients planning pregnancy [44–46].

A study by Korean authors comparing occlusive methods in uterine fibroid treatment was targeted at apoptosis detection. Fibroids extirpated 6 months after occlusive treatment were examined with the following results: while the typical finding in fibroids with acute ischemia caused by UAE was necrosis, in cases after LUAO it was apoptosis [47]. In 8 patients, we also examined fibroids removed following LUAO, which was performed within the previous year and had little effect on both fibroid volume reduction and symptoms. These fibroids were examined with the same technique as in all patients in our study. We did not observe a significant apoptosis rate in these 8 patients, the average AI was 19.3 and thus lower than in women treated with GnRHa. However, in all 8 patients, the occlusive treatment failed and was performed in a longer interval prior to myomectomy (and examination for apoptosis), which could have substantially affected the results.

We can summarize that the three-month pre-operative administration of ulipristal acetate induced natural cell death in uterine fibroids in premenopausal women. On the other hand, in women who had three months of preoperative administration of GnRH analogues or no hormonal therapy, a significantly lower proportion of apoptotic cells in leiomyomas were observed.

## Figures and Tables

**Table 1:** Baseline parameters of the groups of the study.

Type of preoperative treatment	SPRM	GnRHa	No treatment
	(11 patients)	(17 patients)	(10 patients)
Mean age (years)	36.4	33.3	37.9
Mean BMI (kg/m^2^)	24.4	23.0	22.8
Mean diameter of dominant fibroid (mm)	58.3	68.1	60.8
Mean number of myomas (larger than 10 mm)	2.5	2.3	2.6
Mean number of deliveries of patients	0.9	0.6	0.8
Mean interval between last tablet intake/last depot injection and surgery (days)	7.5	35.1	—

BMI: body mass index, GnRHa: gonadoliberin agonist, and SPRM: selective progesterone receptor modulator.

**Table 2:** Apoptotic index (AI) in the subgroups of the study.

Type of preoperative treatment	Number of patients	Mean AI (±SD)	Median of AI	Range of AI	Number of patients with
					AI > 10
SPRM	11	158.9 (±193.2)	96	0–672	9 (81.8%)
GnRHa	17	27.5 (±62.3)	2	0–196	4 (23.5%)
No treatment	10	2.0 (±2.1)	1	0–6	0

AI: apoptotic index, GnRHa: gonadoliberin agonist, and SPRM: selective progesterone receptor modulator.

**Table 3:** Dependence of the number of apoptotic cells on the dose of SPRM.

Daily dose of SPRM	Number of patients	Mean AI (±SD)	Median of AI	Range of AI
Ulipristal 5 mg	6	231.8 (±237.8)	181	26–672
Ulipristal 10 mg	5	71.4 (±71.2)	81	0–173

AI: apoptotic index, SD: standard deviation, and SPRM: selective progesterone receptor modulator.
